# A Multimodal Biomedical Transformer Fusion Network for Disease-Level Rare-Disease-Inheritance Classification Using Ontology-Enriched Text, Metadata, and Gene Associations

**DOI:** 10.3390/biomedicines14071439

**Published:** 2026-06-25

**Authors:** Mahmood A. Mahmood, Khalaf Alsalem

**Affiliations:** Department of Information Systems, College of Computer and Information Sciences, Jouf University, Sakaka 72441, Saudi Arabia; kosalem@ju.edu.sa

**Keywords:** single-cell RNA sequencing, rheumatoid arthritis, multitask deep learning, self-attention network, NK-cDC1 niche identification

## Abstract

**Background/Objectives:** Inheritance classification in rare diseases remains challenging because curated knowledge is incomplete, heterogeneous, and imbalanced across inheritance categories. Disease-level inheritance modeling can support knowledge organization, annotation review, and hypothesis generation in rare-disease resources. This paper introduces RareFusion-Net, a multimodal benchmark framework for disease-level inheritance classification, and evaluates whether integrating ontology-enriched disease text, structured epidemiological metadata, and gene-association information improves prediction in curated rare-disease knowledge bases. RareFusion-Net is intended for knowledge modeling, not individual patient diagnosis. **Methods:** We developed RareFusionBalanced, a gated multimodal fusion model that combines biomedical disease descriptions, structured metadata, and gene-related information using auxiliary supervision. Ontology-enriched disease text was treated as the dominant semantic modality, while tabular and gene modalities were incorporated as complementary evidence when available. Robustness was improved using balanced regularization, selective transformer fine-tuning, dropout, weight decay, label smoothing, early stopping, and prediction aggregation across random seeds. Evaluation included accuracy, macro-F1, micro-F1, macro-AUC, mean average precision, calibration metrics, class-wise analysis, statistical testing, and ablation experiments. **Results:** RareFusionBalanced achieved 0.7382 test accuracy, 0.6284 macro-F1, 0.7382 micro-F1, 0.9183 macro-AUC, and 0.6686 mean average precision. Calibration was favorable, with an expected calibration error of 0.0395 and a Brier-OVR of 0.0528. The multimodal model slightly outperformed TextOnly-TransformerBalanced, but improvement over the best TF-IDF baseline was not statistically significant. Ablation showed ontology-enriched text as the strongest modality, with gene associations adding complementary value. **Conclusions:** RareFusion-Net provides a practical benchmark for ontology-aware rare-disease inheritance modeling. Results suggest selective multimodal benefit while highlighting minority-class difficulty, limited statistical superiority, need for external validation, and improved biological interpretability.

## 1. Introduction

Rare diseases are not common but significant as a group, as they impact up to 3.5% and 5.9% of the total world population, which means hundreds of millions of people worldwide. Although the field of genomics and clinical informatics is continuing to improve, patients with rare diseases continue to experience significant delays in their proper diagnosis before going through multiple handovers, scattered investigations, and misclassification. The systematic reviews of rare diseases within recent years still characterize such a long journey of diagnostic searches as a systems issue within the healthcare field [1].

The primary cause of this challenge is that the evidence in rare diseases is inherently multimodal. Diagnostic reasoning relies not merely on the names of diseases or a written description of the disease but on patterns of inheritance, the organized epidemiological classes, and ontology integrations, as well as the relationship between a gene and a disease. Orphadata is especially topical in this regard since it provides calculable, reusable files of scientific knowledge relating rare diseases to ICD-10, ICD-11, OMIM, UMLS, MONDO, MeSH, MedDRA, GARD, and gene-association databases and is published biannually under an open license. These characteristics enable it to be an appropriate substrate of machine learning and knowledge-sensitive diagnostic modeling [2].

The latest developments in artificial intelligence (AI), specifically machine learning (ML), artificial neural networks (ANNs), and deep learning (DL), have changed contemporary medical research and healthcare analytics considerably. ML facilitates data-driven decision-making based on models that are trained on complex data; ANNs emulate brain-inspired computational structures; and DL expands it by providing multi-layered architectures to extract high-level representations of large-scale and heterogeneous data. These methods have shown to be highly promising in enhancing the accuracy of diagnosis, speed of disease detection, and aid in clinical decision-making in a wide range of medical fields. Furthermore, these AI paradigms are specifically utilized in the biomedical domain, which underscores their capability to process multimodal, complex information, and, therefore, they are especially useful in difficult problems, such as rare-disease analysis, where heterogeneity and sparsity of data play predominant roles [3].

Structured columns in such repositories can be used with traditional machine learning; however, purely tabular models fail to fully represent the semantic links between biomedical codes, disease names, and gene-association phrases. On the other hand, text-based LLM systems not only encode medical terminology and contextual similarity but are optimal predictors in the absence of key evidence that is categorical, numerical, or set-valued. This tension has inspired the development of multimodal AI in medicine, and text, tabular metadata, imaging, time series, genomics, and other streams of evidence are combined into more sophisticated patient or disease representations. Recent reviews always contend that multimodal learning enhances clinical realism and might be more effective than unimodal pipelines in cases when heterogeneous evidence is needed to be combined [4].

The research question of the proposed study is significant since knowledge resources of rare diseases curated by experts offer an organized, biologically informed view of the disease entities, which is challenging to obtain through sparse patient-level data. In these repositories, patterns of inheritance are not described with the help of a single signal but rather manifest themselves in a composite of descriptions of the disease, epidemiological metadata, and data of association between genes. Based on this, the modeling of coarse categories of inheritance based on these resources is not only a technically relevant machine-learning problem, but also a convenient benchmark to study the contribution of complementary biomedical evidence to learning rare diseases. Here, a principled multimodal view of the underlying relational structure of curated rare-disease databases is presented through the combination of text, tabular, and gene-level data.

Pretrained biomedical transformer models offer significant potential for rare-disease informatics because they can encode ontology-enriched disease descriptions, biomedical terminology, and gene-association narratives into dense contextual representations. However, the language component is not used as an autonomous large language model reasoning system. Instead, it serves as a domain-specific text encoder within a multimodal classification framework, where it generates semantic disease embeddings that are subsequently combined with structured epidemiological metadata and gene-level data. In this work, the role of the language branch is to learn biomedical semantic representations rather than perform prompt-based reasoning, generative inference, or autonomous diagnostic decision-making. This distinction is important for properly situating the proposed framework within the broader landscape of artificial intelligence for rare diseases and for avoiding overstatement of the LLM-related component of the approach [5,6,7].

These considerations motivate the present study. Rather than framing the task as LLM-inspired reasoning, we define rare-disease coarse inheritance classification as a multimodal disease-level learning problem based on curated Orphadata knowledge resources. The proposed framework integrates three complementary evidence channels: an ontology-enriched biomedical transformer text branch, a structured epidemiological tabular branch, and an attention-based gene-set branch. In this framework, the transformer provides semantic encoding of disease-related text, while the final prediction is produced through multimodal fusion and multi-task learning. This framing more accurately reflects the methodological contribution of the study and aligns the model with disease-level representation learning rather than autonomous language-model reasoning.

The study design is specifically designed as a disease-level multimodal benchmarking problem, as opposed to a traditional clinical prediction task. The framework utilizes curated rare-disease knowledge in Orphadata, rather than patient-level observations, where patterns of inheritance can be seen because of integrating semantic descriptions, epidemiological metadata, and gene associations. This design enables a regulated assessment of the role of various modalities in the classification of inheritance in realistic sparsity and heterogeneity of data conditions. The suggested arrangement must, therefore, be understood as a representation-learning and knowledge-integration benchmark, intended to inquire into modality contribution and fusion behavior as opposed to actual clinical application.

This work makes four main contributions. First, we introduce a disease-level rare-disease benchmark formulation based on Orphadata 2026 that integrates ontology-aware text, gene associations, prevalence descriptors, and natural-history information into a unified multimodal learning environment. Second, we develop a multimodal fusion framework that incorporates a biomedical transformer text encoder, a structured tabular encoder, and a gene-attention branch for coarse inheritance classification with auxiliary supervision. Third, we reorganize the highly dispersed original inheritance annotations into clinically meaningful coarse classes in order to reduce label sparsity and improve target stability. Fourth, we present a more comprehensive evaluation protocol that includes macro-F1, micro-F1, top-k accuracy, confusion analysis, ROC and precision–recall analysis, calibration assessment, and statistical testing, together with comparisons against simpler and more appropriate baseline models, including text-only linear baselines, tabular machine-learning baselines, and a simple early-fusion multimodal baseline. This broader benchmark setting enables a more rigorous evaluation of the competitiveness of the proposed architecture.

## 2. Literature Review

The most significant problem in informatics of rare diseases is that there are no large, homogenous patient-level datasets with stable labels. It is against this reason that disease-level resources have become the center of rare-disease analytics. Of great significance among them is the Orphanet Rare Disease Ontology (ORDO), which combines rare-disease entities with epidemiological, clinical, and genetic information in a machine-readable format that facilitates interoperability across biomedical systems [8]. Another way in which ORDO enhances the visibility of rare diseases in health information systems is by mapping Orphanet disease entities to major coding schemes and external biomedical terminologies [9,10].

This view that is based on ontology applies directly to machine learning. A single signal does not represent rare diseases; instead, the diseases are represented by a conglomeration of disease names and synonyms, substance associations, phenotypes, prevalence descriptions, and hierarchies. Consequently, disease-level learning over Orphanet-based resources is multimodal in nature. Earlier studies have indicated that ontology-based rare-disease sources can be used to support classification, clustering, semantic similarity analysis, and data harmonization, rendering them not only appropriate in rule-based systems but also in current statistical and representation-learning pipelines [11,12].

Machine-learning studies in rare diseases have steadily increased during the last decade, and diagnosis and prognosis have become the most frequent ones. In a general scoping review of the literature, Schaefer et al. discovered that the majority of the studies took machine learning as an approach to diagnosis or risk prediction, whereas a relative minority of the studies used it in treatment support or translational decision-making. It was also revealed that most studies that deal with rare diseases have very low sample sizes, which diminishes the strength of pure patient-level supervised learning [13].

The findings of the subsequent reviews came up with the same conclusions. Surveys of deep-learning and ML applications in rare genetic and neurological conditions observed that the field is limited by data sparsity, label bias, lack of external validation, and heterogeneity of definitions of the diseases [14,15,16]. All these studies indicate that knowledge-grounded representations can be especially useful in rare-disease AI, as opposed to simple end-to-end supervised learning. This is especially true of the so-called Orphadata-type resources, where disease-level knowledge curation can be more enriched and stable than the associated patient-level observational data [13,14,15,16].

One especially topical research has been based on Orphadata and Orphanet-based data to investigate the internal design of the rare disease space. Frederiksen et al. conducted an in silico epidemiological, genetic, phenotypic, natural-history, and linearization characterization of the rare-disorder spectrum by use of Orphadata data. Their examination revealed that rare disorders are very heterogeneous concerning many dimensions and that no evidence modality is adequate to portray the full spectrum well [17]. This observation has been a very good argument in favor of multimodal learning over the concept of single-source modeling.

This has been advanced by graph-based representation learning. Sanjak et al. created an ontology-enhanced knowledge graph combining rare diseases, genes, phenotypes, small molecules and clustered diseases with node embeddings. Their clustering model has shown that disease representations in the form of embeddings can restore semantically stable clusters and can indicate previously unknown correlations between rare disorders [18]. These cluster-derived structures were subsequently matched to Orphanet classes and were found by Moon et al. to be computational inferences of disease groups that can be mapped to formal taxonomies of rare diseases [19]. Combined, the studies indicate that both semantic organization and machine-learning representation learning can be supported using the disease resources provided by Orphanet.

A semantically based approach to identifying rare diseases using clinical text is another research direction, which uses Orphanet/ORDO as its semantic backbone. Dong et al. suggested an ontology-based weakly supervised method to identify rare diseases with clinical notes, where ORDO-driven labeling can enhance the extraction of rare disease mentions and subsequent normalization. Performance also became an issue in their work as they pointed out that it can significantly decrease when the systems transition to extracting medical concepts not related to a single disease but rather an ontology of the specific disease since the alignment of terminologies is not perfect yet [20].

More recently, a hybrid model was proposed by Wu et al. that integrates ORDO, UMLS, dictionary-based NLP, and large language models to conduct rare-disease phenotyping on clinical note text. Their findings indicate that ontology-constrained LLM pipelines can be more effective than baseline NER and phenotyping systems, which points to the fact that LLMs can be more efficient when provided with a curated vocabulary of rare diseases as opposed to being deployed in an unrestricted free-text environment [21]. Cao et al. proposed a similar direction with AutoRD, which utilized ontology-enhanced LLMs to identify entities and relations to build an automated knowledge graph of rare diseases. Even though AutoRD is more of an information-extraction system, it supports the usefulness of ontology-driven language models in the context of rare-disease informatics [22].

One of the most developed paradigms of rare-disease AI is the one that supports the diagnostic process with phenotypes. Phenotype-driven models were demonstrated earlier in systems like RDAD to be effective at ranking candidate rare diseases and have good behavior on top-k retrieval that can be useful in clinical decision-making [23]. Later frameworks like SHEPHERD used few-shot learning with knowledge graphs of rare diseases to rank consecutive diagnoses of Mendelian diseases to prove that graph-based methods could generalize even in cases when the direct number of cases was small [24].

PhenoDP applied deep learning to prioritize phenotypes as indicators of disease and recommend symptoms, which compared better in quality to previous systems on simulated and real cohorts [25]. These phenotype-based approaches are very pertinent to the diagnosis of rare diseases, and nonetheless, they are not identical to the current work in a critical way: most of them are focused primarily on phenotype information or case-based information, unlike Orphadata, which offers more detailed disease-level multimodal information such as epidemiology, inheritance, external mappings with ontology, and gene sets. This inspires a wider convergence model, which involves multiple evidence lines at an instance compared to utilizing phenotype resemblance by itself [24,25].

The quality of the underlying ontology and mappings is also significant to the success of Orphanet-based AI. Mohtashamian et al. suggested an automated quality-assurance system of ORDO and determined the existence of hundreds of candidates missing hierarchical relations, which demonstrates that the incompleteness of ontology can influence downstream semantic analysis and the quality of learning. This matters since embeddings of diseases, semantic similarity scales, and classification systems all transfer biases or gaps found in the ontology layer on which they rely [26].

On the application level, Faviez et al. showed that supervised learning with semantic phenotyping and rare-disease knowledge resources can be useful in rare-disease detection using EHR data. Their study was highly selective in the identification of patients with NPHP1-related ciliopathy; however, it also highlighted the effects still being experienced because of continued phenotypic recording as well as defective extraction pipelines [27]. Therefore, although the field of rare-disease AI is developing at an impressive pace, strong implementation is yet to be done in consideration of the quality of ontology, systematized disease knowledge, and integration of multimodal evidence [26,27].

The literature summarized in Table 1 suggests that Orphanet-based resources can support ontology engineering, clustering, text phenotyping, disease ranking, and EHR screening. There is, however, a critical gap. Semantic normalization, phenotype-based retrieval, or descriptive structure discovery are also predominantly done using these resources as part of supervised disease-level multimodal prediction; however, not all the studies are based on Orphanet. Specifically, little work has so far been done to learn jointly based on ontology-rich text descriptions, structured epidemiological attributes, and variable-length sets of genes within a single Orphadata-based classification system [17,18,19,20,21,22,24,25,26,27].

The proposed RareFusion-Net is based on formulating Orphadata 2026 as a multimodal disease-level benchmark and on the fusion of three complementary evidence channels: an ontology-aware disease–language–text branch, a tabular disease–epidemiological–natural-history variables branch, and a gene-attention disease-associated gene sets branch. The model, in this manner, builds upon previous representations, retrieval, and extraction-based Orphanet-based rare-disease AI to a more holistic disease-level predictive model.

## 3. Proposed Model

**Figure 1:**
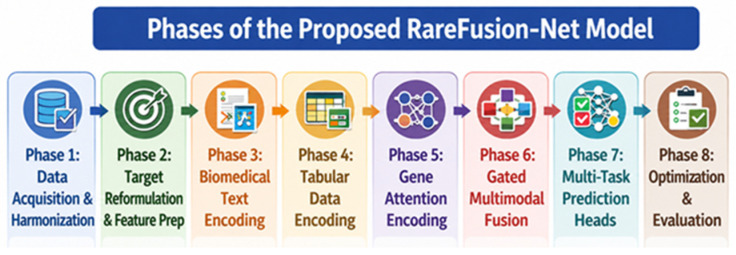
Overall workflow of the proposed RareFusion-Net framework.

Figure 1 presents the general scheme of a workflow of the proposed RareFusion-Net framework of the prediction of rare-disease inheritance with the use of Orphadata 2026. The pipeline starts at the stage of data acquisition and harmonization, in which descriptors of the diseases, gene relations, prevalence data, and natural-history characteristics are unified using the standard OrphaCode identifier. Following preprocessing and target engineering, every disease record is converted into three complementary forms, namely, an ontology-enriched biomedical text prompt, an organized tabular representation, and a gene-set representation. A pretrained biomedical transformer branch, a tabular encoder, and an attention-based gene encoder process these modalities, respectively. The outputting embeddings are then fused using a gated multimodal fusion module, which gradually balances the input of each modality to generate a single disease embedding. The design allows the semantic, epidemiological, and biological information to be maintained by this model and take advantage of their complement to make the prediction of inheritance much more reliable.

The fused representation is learned in a multi-task learning environment, which enhances generalization and representation strength. The first task forecasts the coarse category of inheriting, and two auxiliary tasks identify disorder group and prevalence class, which stimulates the shared embedding to embrace more extensive disease structure and epidemiological background. To decrease label sparsity and enhance clinical interpretability, the fine-grained inheritance annotations are rephrased into clinically meaningful coarse categories, such as autosomal dominant, autosomal recessive, X-linked, mitochondrial, multifactorial, autosomal mixed, and unknown/other. By doing so, RareFusion-Net goes beyond unimodal learning directly modeling the ontology-rich and heterogeneous nature of rare-disease knowledge bases to offer an effective multimodal model that is useful in disease-level classification of curated Orphadata knowledge bases.

### 3.1. Phase 1: Data Acquisition and Harmonization

The initial step of the intended model standardizes the heterogeneous Orphadata files into a single disease-level datum as shown in Algorithm 1. The initial repository holds numerous tables, one of which is the core disease data, which is associated with genes, the prevalence data, and natural-history data. The files are all connected by the OrphaCode identifier, though they each represent a different facet of knowledge about rare diseases. This stage is then aimed at the integration of these distributed resources into a unified multimodal table, which can be fed into the downstream model.

Through the process, repeated records are added together and converted into summaries on the disease levels. In the case of gene associations, the framework gathers special gene symbols and descriptive text of gene associations for each disease. To obtain information on prevalence, it calculates representative values of prevalence type, prevalence class, geographic scope, and average numeric prevalence. In the case of natural-history data, it derives the prevailing age-of-onset and inheritance characteristics. The missing values are coded into a single representation of a placeholder to ensure that the model can differentiate between the missing information and valid observations. This phase of integration is essential since it transforms the disjointed biomedical knowledge into an integrated multimodal sample representation.

The dataset used in this paper was constructed from a Kaggle-hosted package of the February 2026 Orphadata files, rather than through direct online querying of the Orphanet infrastructure during model training. Therefore, the resulting benchmark should be understood as a reproducible derived cohort assembled from multiple distributed source tables, rather than as a single native disease-level dataset. The harmonization process was carried out programmatically through deterministic aggregation and merge operations using OrphaCode as the primary key, with no manual case-by-case label editing beyond the explicitly defined preprocessing and target-mapping rules.
**Algorithm 1: Disease-Level Data Harmonization**Input: Rare disease information table I, Gene association table G, Prevalence table P, Natural-history table N Output: Unified disease-level multimodal table D Steps: 1. Initialize D from the disease information table I. 2. Group G by OrphaCode and aggregate:             a. unique gene symbols,             b. gene names,             c. association types,             d. association status. 3. Group P by OrphaCode and aggregate:             a. dominant prevalence type,             b. dominant prevalence qualification,             c. dominant prevalence class,             d. dominant geographic prevalence scope,             e. mean prevalence value,             f. number of prevalence records. 4. Group N by OrphaCode and aggregate:             a. dominant age of onset,             b. dominant inheritance descriptor. 5. Merge all aggregated tables with D using OrphaCode. 6. Fill missing categorical values with a unified placeholder token. 7. Fill missing numerical values with zero or aggregated defaults. 8. Return the final disease-level multimodal table D.

### 3.2. Phase 2: Target Reformulation and Feature Preparation

In the second step, the raw inheritance target was reformulated into a clinically interpretable coarse-label space, and the multimodal inputs were prepared accordingly. The original Orphadata inheritance field contains fragmented and heterogeneous descriptors, including explicit inheritance modes, mixed patterns, unknown values, non-applicable entries, and sparse edge cases. Direct modeling of these raw labels would result in a long-tailed multiclass problem with limited statistical robustness. To address this issue, as shown in Algorithm 2 the raw inheritance descriptors were mapped into seven broad categories according to predefined rules: autosomal dominant was assigned to Autosomal Dominant; autosomal recessive to Autosomal Recessive; descriptors containing both autosomal dominant and autosomal recessive to Autosomal Mixed; descriptors containing x-linked to X-linked; descriptors containing mitochondrial to Mitochondrial; descriptors containing multigenic or multifactorial to Multifactorial; and descriptors such as missing, no data available, unknown, and not applicable, along with any remaining unmatched cases, to Unknown/Other. Semi-dominant entries were additionally grouped under Autosomal Dominant, as shown in Table 2. This explicit reformulation reduces label fragmentation while preserving clinically meaningful inheritance structure and ensuring fully reproducible target construction.

Simultaneously, the framework forms three input modalities. A disease-level textual prompt is first formed based on the name of the disease, ontology codes, and gene-association descriptors. Second, tabular branching extracts are structured categorically and numerical variables. Third, the gene branch is prepared with a variable number of associated genes. By this step, the noise of labels is minimized, and the informativeness of inputs is maximized, thus forming the precursor of the next multimodal encoding procedure.
**Algorithm 2: Target Engineering and Multimodal Input Construction**Input: Unified disease-level table D Output: Refined table D*, Main target y_main, Text features X_text, Tabular features X_tab, Gene-set features X_gene Steps: 1. For each disease record in D:             a. read the raw inheritance descriptor,             b. map it into a coarse inheritance category. 2. Remove classes whose support is below a minimum threshold if needed. 3. Construct X_text by concatenating:             a. disease name,             b. ontology mappings,             c. gene association descriptions. 4. Construct X_tab from:             a. categorical metadata,             b. numerical epidemiological features. 5. Construct X_gene as the set of associated gene symbols. 6. Define auxiliary labels:             a. disorder group,             b. prevalence class. 7. Return D*, y_main, X_text, X_tab, and X_gene.

### 3.3. Phase 3: Biomedical Text Encoding

The text branch is there to be able to represent the semantic and ontology-sensitive representation of each disease as shown in Algorithm 3. The proposed textual prompt has a more elaborate structure than simply the disease name but contains cross-references to ICD-10, ICD-11, OMIM, MONDO, UMLS, MeSH, MedDRA, GARD, and gene-association descriptions. This prompt is acquired with a pretrained biomedical transformer, allowing the network to learn contextual domain-relevant embeddings that capture biomedical relationships and rare-disease terms.

Mean pooling of the transformer contextual token embeddings is obtained to obtain the final text representation, which is then projected into the shared latent space. Through this branch, the model can encode fine-grained semantic relationships that otherwise would be hard to maintain in manually engineered features. This is particularly applicable in cases where there are informative ontology descriptors or other well-defined descriptions of the association between diseases and genes.
**Algorithm 3: Biomedical Text Encoding**Input: Text prompt X_text for each disease, Biomedical transformer encoder T Output: Disease-level text representation Z_text Steps: 1. Tokenize each text prompt X_text. 2. Feed tokenized text into transformer encoder T. 3. Obtain contextual token embeddings H. 4. Apply mean pooling over H to generate a fixed-length vector v_text. 5. Pass v_text through a projection layer. 6. Return projected text representation Z_text.

### 3.4. Phase 4: Tabular Data Encoding

The tabular branch models disease-level metadata that complement the ontology-enriched text input. These features include explicit availability indicators for key structured information sources as shown in Algorithm 4. Missing categorical values are represented using a common placeholder token (missing), and category vocabularies are constructed from the training split with reserved [PAD] and [UNK] tokens. Each categorical field is mapped to a learnable embedding of dimension 24. Numerical variables are imputed with zero where necessary and standardized using a StandardScaler fitted exclusively on the training set. The resulting categorical embeddings and standardized numerical features are concatenated and fed into a multilayer perceptron with a hidden dimension of 160 to generate the final tabular representation. This design allows the tabular branch to retain explicit epidemiological regularities while remaining robust to sparsity and incomplete metadata.
**Algorithm 4: Structured Metadata Encoding**Input: Categorical metadata X_cat, Numerical metadata X_num Output: Structured representation Z_tab Steps: 1. Encode each categorical variable using an embedding layer. 2. Normalize numerical variables using feature standardization. 3. Concatenate embedded categorical vectors with normalized numerical features. 4. Pass the concatenated vector through a multilayer perceptron. 5. Return the tabular latent representation Z_tab.

### 3.5. Phase 5: Gene Attention Encoding

The gene branch models disease-associated gene sets as symbolic inputs. In the current implementation as shown in Algorithm 5, each gene symbol is encoded as an integer index derived from the training vocabulary, with reserved [PAD] and [UNK] tokens used for padding and previously unseen genes, respectively. These indexed symbols are mapped to learnable gene embeddings of dimension 160, which are randomly initialized and optimized jointly with the rest of the network rather than initialized from an external pretrained gene-embedding resource. To manage variability in gene-set size, a maximum sequence length is imposed: sets containing more than 16 genes are truncated, whereas shorter sets are padded with the [PAD] token. Fully padded sequences correspond to diseases for which no associated gene symbols are currently available. Rather than introducing invalid values, these sequences explicitly represent the absence of molecular annotations. An attention mechanism is then applied over the embedded gene set to assign adaptive importance weights to individual genes, and the resulting weighted sum is projected into a shared 256-dimensional fusion space. This design enables the model to capture gene-level disease structure while remaining robust to substantial variation in gene-set cardinality and to missing molecular annotations.
**Algorithm 5: Gene-Set Attention Encoding**Input: Gene symbol set X_gene for each disease Output: Gene representation Z_gene Steps: 1. Build a training-derived gene vocabulary with reserved [PAD] and [UNK] tokens. 2. Convert each disease-associated gene symbol into an integer index. 3. Retain up to 16 genes per disease and pad shorter sets with [PAD]. 4. Represent diseases with no genes as fully padded gene sequences. 5. Map indexed genes into learnable embedding vectors. 6. Compute an attention score for each embedded gene and mask padded positions. 7. Normalize attention scores using softmax. 8. Compute the attention-weighted pooled gene representation. 9. Pass the pooled vector through a projection layer.10. Return the final gene representation $Z_{gene}$.

### 3.6. Phase 6: Gated Multimodal Fusion

The proposed model combines the three modality-specific representations as shown in Algorithm 6 after their extraction, with the help of a gated fusion mechanism. In the case of simple concatenation of features, it is assumed that there is the same reliability of all the modalities, which is unrealistic in rare-disease data since not all diseases have the same completeness. There are those with rich gene annotations and poor prevalence information and others with better structured metadata and poor association text. To curb this, the model is trained to learn different gates of the text, tabular, and gene branches.

A gate generates an adaptive weight vector that scales the associated modality representation, followed by fusion. The reweighted features are further concatenated and fed through a common fusion network to give the final disease embedding. This process enhances the level of robustness since the model can focus on the most informative modality of each individual disease sample.
**Algorithm 6: Gated Multimodal Fusion**Input: Text representation Z_text, Tabular representation Z_tab, Gene representation Z_gene Output: Shared fused representation Z_fused Steps: 1. Compute text gate g_text from Z_text. 2. Compute tabular gate g_tab from Z_tab. 3. Compute gene gate g_gene from Z_gene. 4. Reweight each modality:             a. Z_text’ = g_text ⊙ Z_text             b. Z_tab’  = g_tab  ⊙ Z_tab             c. Z_gene’ = g_gene ⊙ Z_gene 5. Concatenate Z_text’, Z_tab’, and Z_gene’. 6. Pass the concatenated vector through a shared fusion network. 7. Return the shared fused representation Z_fused.

### 3.7. Phase 7: Multi-Task Prediction Heads

The framework as shown in Algorithm 7 includes one primary task and two auxiliary tasks built upon a shared fused representation. The primary output predicts the coarse inheritance category, whereas the auxiliary heads predict disorder group and prevalence class. These auxiliary targets were introduced to encourage the shared representation to capture broader disease structure beyond the inheritance label alone. Conceptually, such supervision may enhance representation quality in rare-disease learning by regularizing the latent space and exposing the model to related disease-level signals.
**Algorithm 7: Multi-Task Prediction**Input: Shared fused representation Z_fused Output: Main prediction y_main_hat, Auxiliary prediction y_aux1_hat, Auxiliary prediction y_aux2_hat Steps: 1. Feed Z_fused into the main classifier head to obtain y_main_hat. 2. Feed Z_fused into auxiliary head 1 to obtain y_aux1_hat. 3. Feed Z_fused into auxiliary head 2 to obtain y_aux2_hat. 4. Return all task-specific outputs.

### 3.8. Phase 8: Optimization and Evaluation

PyTorch version 2.9.0 was used to implement the model, with a pretrained biomedical transformer serving as the text encoder. Disease prompts were tokenized to a maximum length of 128 tokens. The text branch learned pooled transformer-based representations, which were projected into a shared latent space of dimension 256. The tabular branch learned categorical embeddings of dimension 24 and used a hidden dimension of 160, while the gene branch learned embeddings of dimension 160 and projected them into the same 256-dimensional shared space. The model was trained for up to 50 epochs using AdamW, with a learning rate of $1.3\times 10^{-5}$ for transformer parameters and $7\times 10^{-4}$ for the remaining network components, a weight decay of $1\times 10^{-4}$, dropout of 0.25, gradient clipping at 1.0, and early stopping with a patience of 9 epochs. Class imbalance was addressed using effective-number class weights and a weighted sampler. Optimization employed an LDAM-based loss schedule for the main task, while the auxiliary tasks used cross-entropy loss with label smoothing of 0.01. The overall objective was defined as a weighted combination of the main and auxiliary losses, with weights of 1.0 for inheritance prediction, 0.12 for disorder-group prediction, and 0.08 for prevalence-class prediction. In addition, an exponential moving average with a decay of 0.995 and multi-seed training were used to improve training stability and generalization.

During inference as shown in Algorithm 8, a disease record is passed through the three modality branches, the gated fusion module, and the prediction heads. The model outputs the predicted coarse inheritance category together with class probabilities, enabling both top-1 and top-k evaluation. This design supports not only strict classification but also ranking-based decision support, which may be useful when multiple inheritance hypotheses are clinically plausible.

We also reported raw reliability metrics and applied post hoc calibration using temperature scaling. Validation-set probabilities were used to fit a scalar temperature parameter by minimizing the negative log-likelihood, and the learned temperature was then applied to calibrate the test-set logits before computing class probabilities. Expected calibration error and the average one-vs-rest Brier score were used to evaluate calibration quality before and after scaling.
**Algorithm 8: Training and Inference Procedure**Input: Training dataset with text, tabular, gene, and task labels, RareFusion-Net model Output: Trained model and predicted inheritance probabilities Training Steps: 1. Sample a mini-batch of diseases. 2. Encode text, tabular, and gene modalities. 3. Fuse the modality representations using gated fusion. 4. Produce main and auxiliary predictions. 5. Compute:             a. weighted main loss,             b. auxiliary loss 1,             c. auxiliary loss 2. 6. Sum losses using predefined task weights. 7. Backpropagate gradients and update model parameters. 8. Repeat until convergence or early stopping. Inference Steps: 1. Input a disease record. 2. Compute text, tabular, and gene representations. 3. Fuse representations using learned gates. 4. Obtain class logits from the main head. 5. Apply softmax to generate inheritance probabilities. 6. Return top predicted class and ranked candidate classes.

### 3.9. Implementation Details

Each experiment was conducted with three random seeds (42, 52, and 62), and prediction was obtained by averaging the predicted probabilities across the three runs. The transformer encoder was kept frozen during the first two epochs and was then optimized jointly with the remaining branches. After epoch 6, dynamic reweighting was applied to the main task, and an exponential moving average with a decay factor of 0.995 was used throughout training. The primary classifier employed a multi-sample dropout head with five dropout samples, while modality dropout was set to 0.08 during multimodal training. These implementation choices were adopted to improve optimization stability, reduce overfitting, and enhance robustness to the severe class imbalance in the rare-disease benchmark.

## 4. Experimental Results and Discussion

### 4.1. Dataset

The experiments were conducted using the February 2026 release of the Orphadata rare-disease resource hosted on Kaggle, which served as the data source for this study. The benchmark was constructed from five CSV files that were harmonized programmatically at the disease level using the shared OrphaCode identifier. Specifically, gene records were aggregated into disease-level gene sets and association summaries, prevalence records were reduced to representative prevalence descriptors and numerical summaries, and natural-history records were summarized into dominant age-of-onset and inheritance descriptors. Additional derived variables were subsequently generated to support multimodal learning, including gene counts, counts of recorded prevalence entries, log-transformed numerical features, and data-availability indicators. The analysis was therefore performed on a derived disease-level modeling cohort rather than on an unprocessed raw table. All preprocessing steps were implemented automatically and reproducibly: rows with missing main inheritance labels were excluded, raw inheritance descriptors were mapped into predefined coarse classes, and low-support classes were filtered using an explicit minimum-count threshold. No additional harmonization or target-engineering rules were introduced beyond those explicitly described, and no manual case-by-case relabeling was performed. The final benchmark produced by this curation pipeline comprised 6238 disease records, partitioned into 4366 training, 936 validation, and 936 test samples [6].

Table 3 shows that the final benchmark is markedly imbalanced, with Autosomal Recessive, Unknown/Other, and Autosomal Dominant accounting for the largest numbers of samples, whereas Autosomal Mixed and Mitochondrial are represented by only a few cases. A stratified train/validation/test split was used to preserve the overall class distribution across subsets as closely as possible. However, under such severe imbalance, a single split cannot provide fully stable conclusions for all classes. This limitation is especially important for the smallest categories, whose performance estimates may vary considerably across partitions. Accordingly, the reported holdout results should not be regarded as the sole basis for stability assessment, but rather as one component of a broader evaluation framework that also includes repeated stratified analysis or cross-validated baseline comparisons. Even so, the benchmark remains valuable because it reflects the realistic class imbalance of curated rare-disease resources while still supporting controlled evaluation through class weighting, imbalance-aware training, and multimodal comparison.

The structured branch comprised five categorical and ten numerical disease-level variables, including prevalence descriptors, age-of-onset information, gene-related count features, and explicit availability indicators for prevalence-related data. Because the final class distribution is directly determined by the inheritance-collapsing rules, the raw-to-coarse target mapping is reported explicitly in the Methods section to ensure reproducibility.

### 4.2. Results

To place the proposed framework in a more meaningful comparative context, we also evaluated several stronger baseline models tailored to the available modalities. These included text-only baselines based on TF–IDF features with logistic regression and linear SVM, tabular baselines based on random forest and histogram-based gradient boosting, and a simple early-fusion multimodal baseline that combined reduced text features with structured variables. This broader baseline setting provides a more stringent reference for assessing the potential advantages of the proposed architecture over simpler and more conventional approaches.

To strengthen the comparative analysis, we complemented the single holdout split with repeated stratified evaluation of the simpler baseline models. This additional analysis provides a broader view of performance variability across data partitions and is particularly important in the present benchmark because of the strong class imbalance and the limited support for some inheritance categories. Accordingly, the study conclusions are based not only on a single train/validation/test split, but also on repeated partition-based evidence from key baseline models.

Table 4 shows the performance of various classical holdout baselines in the coarse inheritance classification task. The TFIDF_LinearSVM baseline performed best in terms of overall classification accuracy, macro-F1, micro-F1, and mAP scores, which were 0.7340, 0.6062, 0.7340, and 0.6244, respectively. This means that the most discriminative signal for predicting inheritance categories is in the form of ontology-enriched textual information. The TFIDF_LogReg model showed lower accuracy (0.7418) and macro-F1 (0.6880) than LinearSVM, but it has higher macro-AUC (0.8720) and a better Brier score (0.0704) than LinearSVM, which would demonstrate better ranking and probability-quality behavior than LinearSVM with the weaker hard-label classification performance. The EarlyFusion_LogReg model failed to outperform the text-only TF-IDF models, suggesting that early fusion was not an effective way of leveraging the extra tabular or gene-related signals. The tabular-only models, particularly Tabular_RandomForest, on the other hand, had much poorer performance, in line with previous findings that structured metadata alone is not enough for this task.

**Figure 2:**
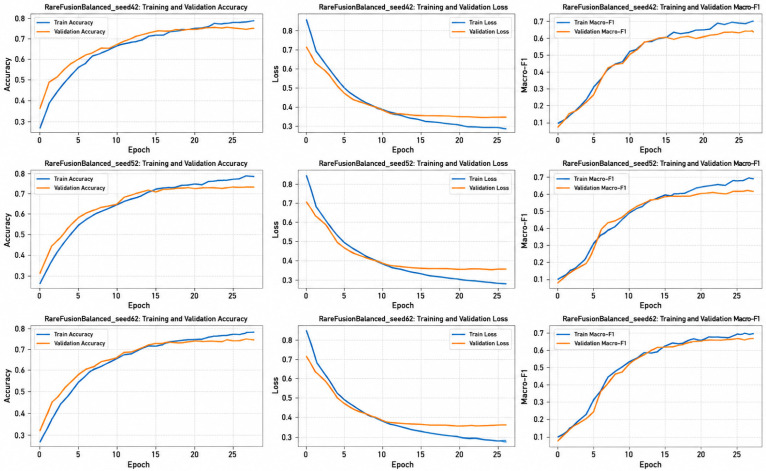
Accuracy and loss training and validation for proposed model.

The training and validation behavior for three random seeds (seed 42, 52, and 62) are shown in Figure 2. For every seed, the figure displays accuracy, loss and macro-F1 curves, offering a comprehensive look at the optimization and stability of generalization of the model. As seen in the accuracy curves, the model’s learning steadily improves in the early epochs, and also slowly stabilizes, suggesting it gradually learns discriminative inheritance-related patterns. Likewise, there is a steady drop in both the loss curves for the training and validation sets, with the validation set becoming less divergent after the middle epochs. The macro-F1 curves also show improvement over epochs, especially since they are a representation of the model’s behavior on both majority and minority inheritance classes. A small train–validation gap is observed in the later epochs, but the gap between train and validation loss is not large and does not experience sharp divergence: generalization behavior is acceptable, albeit with mild overfitting.

Table 5 shows the classification accuracy of the proposed model in the coarse inheritance classification task for each class. The accuracy of the model was 0.7382, meaning that about 74% of the test samples were correctly classified. Autosomal Recessive had the highest precision (0.8042), recall (0.8313), and F1-score (0.8175), indicating that the model can effectively distinguish the inheritance category of Autosomal Recessive, which is one of the most well-represented classes. In the same way, Unknown/Other and X-linked classes also had good F1 scores of 0.8128 and 0.7939, respectively, which demonstrates a high recognition rate for these classes. The performance of Autosomal Dominant was also decent, with an F1 score of 0.6878, although there were still some overlaps with other autosomal classes. Lighter performance could be seen in Autosomal Mixed and Multifactorial with F1-scores of 0.3218 and 0.4478, respectively. Such lower values indicate that the model still has some difficulties distinguishing between the different inheritance categories, as well as overlapping categories. The F1 score of the Mitochondrial class is 0.5000, which should be taken with a grain of salt because this class is only sparsely supported in the test set. Overall, the table indicates that the proposed model is fairly accurate for the large inheritance classes, but not so accurate for the rare, low-support, and biologically heterogeneous inheritance classes.

**Figure 3:**
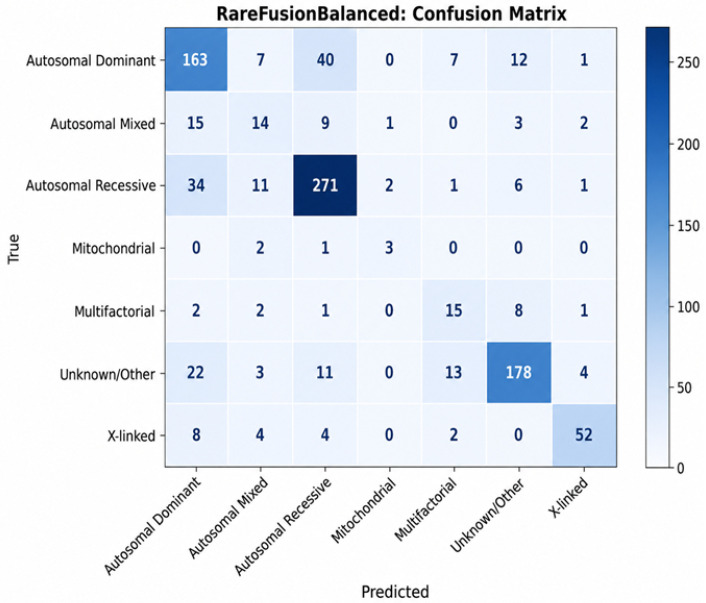
Confusion Matrix of proposed model.

The confusion matrix of the proposed RareFusionBalanced model for the coarse inheritance classification task is shown in Figure 3. Opposite the diagonal entries are the numbers of correctly classified samples, and off the diagonal are the numbers of samples misclassified among the categories of inheritance. The figures for the major classes are also good—271 samples were correctly classified for Autosomal Recessive, 178 for Unknown/Other, 163 for Autosomal Dominant, and 52 for X-linked. The results showed that the model can capture the dominant inheritance pattern sufficiently with the support of the class, but there is some overlap and/or confusion in the categories that are biologically related. For instance, when disease-level descriptions have overlapping genetic or clinical signals, the distinction between autosomal inheritance modes may be difficult to make, and so may be misclassifications such as Autosomal Dominant being misclassified as Autosomal Recessive (40 cases) and Autosomal Recessive being misclassified as Autosomal Dominant (34 cases). The minority classes (Autosomal Mixed, Multifactorial, and Mitochondrial) also exhibit poorer results, as the number of predictions made correctly is not large enough to be clearly visible in this matrix. The Mitochondrial class only contains six test samples, so the diagonal value of three predictions should be taken with a pinch of salt. Overall, Figure 3 validates the suitability of the proposed model for well-represented categories of inheritance, but its suitability is lower for rare and heterogeneous categories of inheritance, pointing out the effects of class imbalance on rare-disease-inheritance classification.

**Figure 4:**
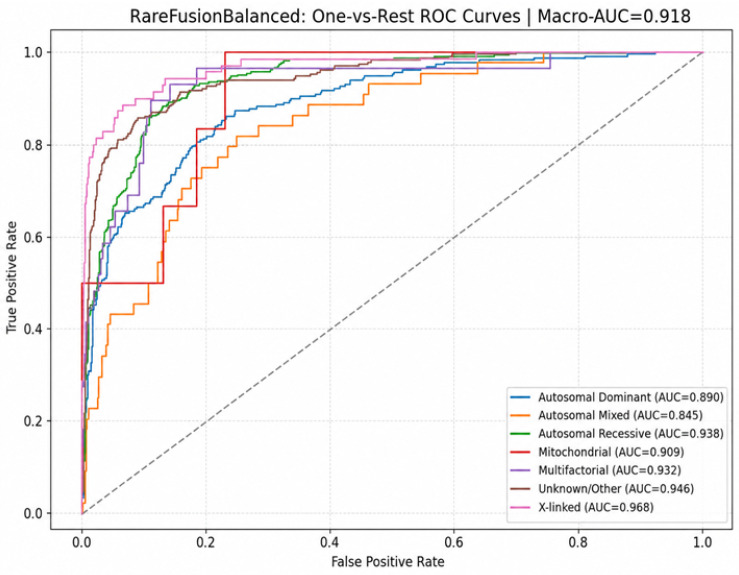
Macro AUC for proposed model.

Figure 4 shows the one-vs-rest ROC curves of the proposed RareFusionBalanced model for the seven coarse inheritance classes. Overall model discriminatory performance was good with a macro-AUC of 0.918 across all inheritance groups. The ROC curves are always located above the diagonal reference line, indicating that the model’s performance is significantly better than a random classification. The highest AUC was for X-linked inheritance (0.968), followed by Unknown/Other (0.946), Autosomal Recessive (0.938), and Multifactorial (0.932). In addition, good AUC values were obtained for both the Mitochondrial (0.909) and the Autosomal Dominant (0.890) models. The lowest AUC was found for Autosomal Mixed (0.845), indicating that this class still seems to be more complex to distinguish from the other classes of autosomal inheritance. Overall, the proposed model shows high macro-AUC, indicating the strong ranking and class-separation ability despite some minor and overlapping classes.

**Figure 5:**
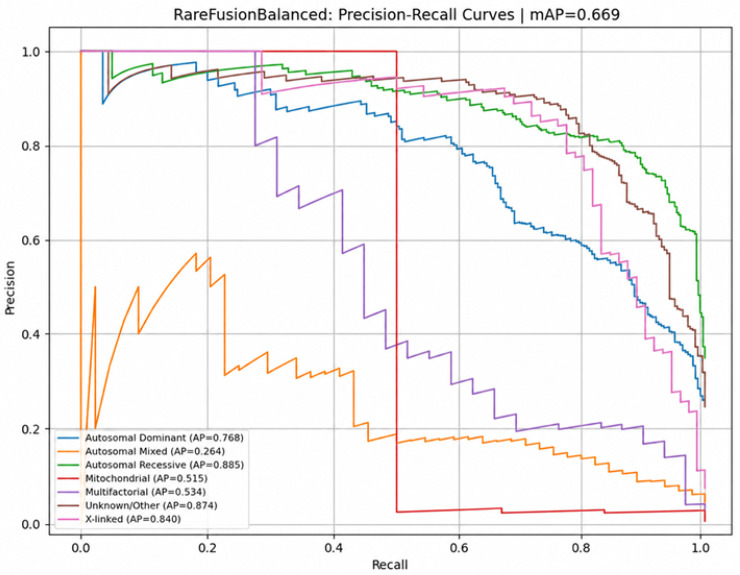
mAP curve for proposed model.

The mean average Precision (mAP) scores of the proposed model (RareFusionBalanced) for the seven coarse inheritance classes are shown in the form of a precision–recall (PR) curve in Figure 5, along with the mean Average Precision (mAP) of the overall model, which was 0.669. This figure assesses the model based on precision–recall curve aspects, which is useful for imbalanced classification problems, as it measures the model’s ability to maintain high precision at high recall. Overall, the model had the highest AP for Autosomal Recessive (0.885), followed by Unknown/Other (0.874) and X-linked (0.840), which suggests that these classes are determined with relatively high accuracy for a wide range of recall values. Autosomal Dominant also presented moderately good performance, obtaining an AP value of 0.768, suggesting that this huge inheritance class can be captured effectively with the model. The performance was poorer for the classes of Multifactorial (AP = 0.534), Mitochondrial (AP = 0.515), and especially Autosomal Mixed (AP = 0.264), which indicates that these classes are still harder to handle due to the small sample sizes, class imbalance, and overlap with other inheritance classes. The relatively irregular PR curves of the minority groups also indicate the instability that is generally found when the number of positive samples is small. Overall, the proposed model shows good precision–recall performance in the better-represented inheritance categories, and it is not very precise in rare and heterogeneous categories, as observed in Figure 5, but it is still meaningful with a moderate overall mAP value.

**Figure 6:**
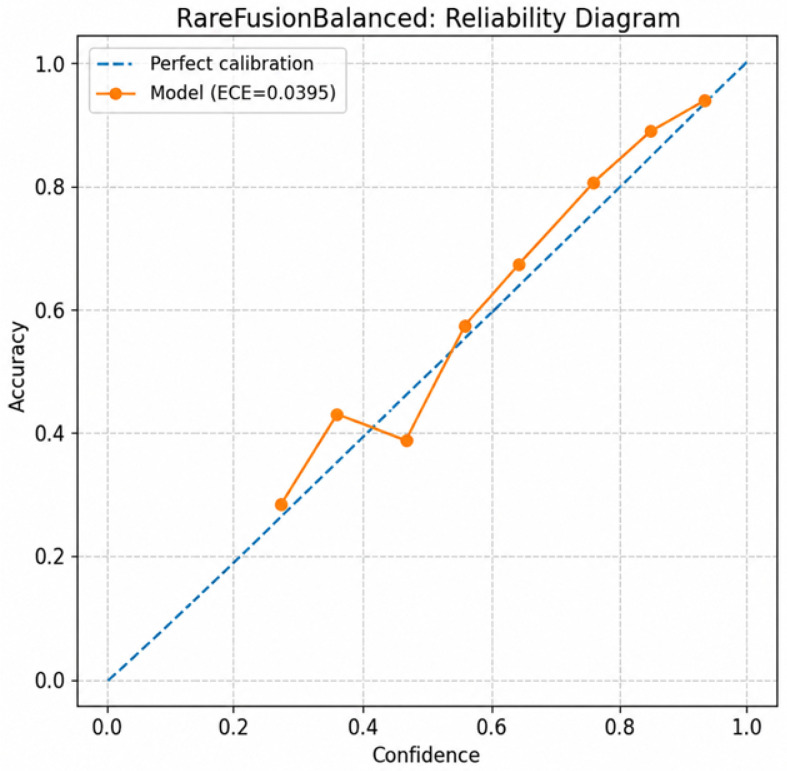
Reliability for proposed model.

Figure 6 shows the accuracy of the predicted confidence scores in comparison to the actual classification accuracy; a reliability diagram is provided for the proposed RareFusionBalanced model. The dotted diagonal line shows ideal calibration, where the expected accuracy matches the observed accuracy. The orange curve represents the calibration behavior of the model and has an expected calibration error (ECE) of 0.0395. The curve is close to the diagonal line for most of the confidence intervals, suggesting that the model is well calibrated in terms of the predicted probabilities. Below and within the middle ranges there are some small deviations, which might be interpreted as the estimation being too high or too low in some probability bins. Model predictions are, however, very close to the ideal calibration line in the higher confidence ranges, suggesting that high-confidence predictions tend to be very reliable. In summary, the low ECE value and the good agreement with the diagonal line show that the proposed model yields reliable and consistent probability estimates, which is crucial for rare-disease-inheritance classification for which calibrated confidence can assist in the expert review and decision-making process.

The stability of the proposed model is presented in Table 6 for three different random seeds: 42, 52, and 62. The results indicate that the test accuracy for the three runs performed is not strongly dependent on a single random initialization of the model, as the model’s overall predictive performance is not drastically affected by this variation. Seed 62 provided the highest test accuracy (0.7447), and seed 42 had the highest test macro-F1 (0.6224), resulting in slightly better class-balanced prediction for that run. The validation results are also found to be stable, ranging from 0.6302 to 0.6839 for validation macro-F1 and 0.7254 to 0.7575 for validation accuracy. Also, the gate values agree across seeds, with the text gate set to 1.0000, as the ontology-enriched disease text is the prevalent reference modality, and the tabular and gene gates are close to each other as the runs progress, between 0.41 and 0.43 and 0.39 and 0.40 respectively. This suggests that tabular metadata and gene associations present complementary, but more limited, information. In summary, Table 6 shows that the proposed model has good levels of stability at the seed point and small variations in performances, although the differences in macro-F1 indicate that the prediction of minority classes is somewhat sensitive to the initialization process.

The performance of the proposed model for different prediction setups is shown in Table 7. The proposed model not only had the highest accuracy but also had the best macro-F1 score (0.6284) and micro-F1 score (0.7489). The accuracy was slightly reduced when the temperature scaling was applied (0.7329) and the macro-F1 score was reduced from 0.6284 to 0.5948, suggesting that the calibration process has not improved the final class assignments and may have even degraded the class-balanced performance. The bias tuning after temperature scaling did not give any extra benefit for this dataset, since similar results were obtained with bias tuning alone. The above results show that the initial proposed model is the best prediction. Even if calibration analysis does not improve discrete classification metrics such as accuracy or F1-score, however, it can be useful in assessing the reliability of confidence.

The statistical assessment of the proposed model is given in Table 8, which also provides bootstrap confidence intervals, comparisons of the proposed model with the baseline models, and calibration measures. The proposed model obtained an accuracy of 0.7382 and a micro-F1 of 0.7382 on the test set with a 95% confidence interval of [0.7147, 0.7714], which is a relatively stable overall performance on the test set. In the case of the bootstrap macro-F1, the value was 0.6284 with a wider confidence interval of [0.5513, 0.6813]—a higher level of uncertainty for class-balanced performance, as there is an imbalance among the various types of inheritance, and minority classes have limited support.

The statistic against the majority baseline was 283.6688 with a *p*-value of 0.0000—these results were highly significant, indicating that the proposed model achieves significantly better performance than a simple majority classifier. The comparison was not statistically significant, however, as indicated by the McNemar *p*-value of 0.5667 with the strongest baseline TFIDF_LinearSVM. Likewise, the paired bootstrap macro-F1 gain for TFIDF_LinearSVM was 0.0196 with a 95% CI of [−0.0246, 0.0655]. This means that although the proposed model did improve slightly in numbers from the best text baseline, these improvements are to be taken with a grain of salt and cannot be deemed statistically significant.

After temperature scaling, the calibration metrics have good probability reliability, with a mean one-vs-rest Brier score of 0.0528 and an expected calibration error of 0.0395. The values indicate that the model’s predictions are close to the real world. Overall, it is clear from Table 8 that the proposed model outperforms the majority baseline model and makes well-calibrated predictions, and that its performance over the best TF-IDF baseline model is still not enough to be statistically significant.

Table 9 gives some sample predictions for the main coarse inheritance classification task, based on the proposed model. The examples demonstrate that the model does a good job identifying multiple well-represented and semantically clear categories of inheritance, including autosomal recessive and X-linked disorders. For example, diseases like osteopenia-intellectual disability-sparse hair syndrome were correctly defined as autosomal recessive, and diseases such as arthrogryposis multiplex congenita were correctly classified as X-linked. Correct predictions are also included in the table for the unknown/other and Autosomal Dominant cases, showing that several key inheritance patterns can be captured from the disease-level text, prevalence descriptors, and gene-related information. The other examples in this task, however, are wrong to show the other difficulties in the task. The model misclassified cases of Barber–Say syndrome as Autosomal Dominant rather than Autosomal Mixed and some cases of Autosomal Dominant such as Larynx atresia, ADAR-related hereditary spastic paraplegia, and SHORT syndrome as Unknown/Other and Autosomal Recessive. The errors reflect the difficulties of the model in dealing with the following: Ambiguous or overlapping inheritance descriptions, missing metadata, and biologically heterogeneous categories. The overall impression from the sample predictions is that the proposed model was successful for many clear inheritance patterns but was less successful in the categories that fall on the boundary and those where it was not supported as much.

### 4.3. Ablation Study

The ablation study is shown in Table 10 to assess the contribution of each modality in the proposed framework. The proposed model outperformed all other models in terms of test accuracy (0.7382), test macro-F1 (0.6284), and test micro-F1 (0.7382). This means that the best performance for the overall accuracy/balanced prediction of the classes was achieved when using the full multimodal configuration. The Text_Gene variant also yielded very close results, with the test accuracy of 0.7372, the test macro-F1 of 0.6224, and the highest validation macro-F1 of 0.6716. This implies that the information contained in gene associations is useful when combined with ontology-enriched disease text. The Text_only model is also quite competitive, with its test accuracy score of 0.7318 and test macro-F1 score of 0.6127, demonstrating that textual disease descriptions are the most important predictive modality for this task. However, the Text_Tab variant had marginally poorer test performance, suggesting that gene information is more strongly associated than structured tabular metadata. Tab_Gene gave the lowest accuracy (0.5321) and macro-F1 (0.3159) in the tests, indicating that the non-text modalities are not enough to accurately predict the inheritance class. Overall, the ablation results show that the proposed multimodal design is most effective and that text is the primary discriminating source and gene information is the most complementary.

**Figure 7:**
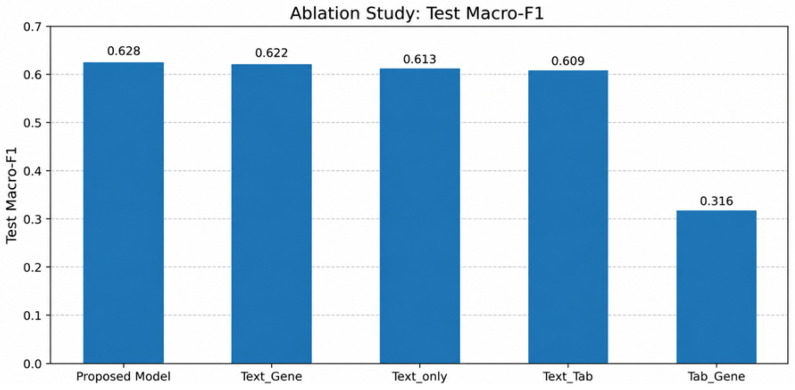
Macro-F1 ablation study.

The ablation study results are demonstrated in Figure 7, where the effect of various modality combinations on the performance of the proposed model is shown in terms of class-balanced performance. The proposed model, text + tabular metadata + gene information, demonstrated the highest test macro-F1 classification score of 0.628, thus validating the combination of the full multimodal model to yield the best-balanced classification results. The Text_Gene variant yielded a very close macro-F1 score of 0.622, showing that gene associations have meaningful complementary information in combination with ontology-enriched text. The Text_only model was also competitive, with a macro-F1 of 0.613, further demonstrating that text at the disease level is the most informative individual modality for inheritance classification. The Text_Tab variant had slightly lower macro-F1 (0.609), indicating that the contribution of tabular metadata is less than the contribution of gene information. The Tab_Gene variant, however, achieved the lowest macro-F1 score of 0.316, indicating that the non-text modalities were not enough for making reliable class-balanced predictions. The overall figure shows that the proposed multimodal design is the most successful model in terms of macro-F1 performance, with text being the most important predictive source and gene-level information the most useful complementary source of information.

**Figure 8:**
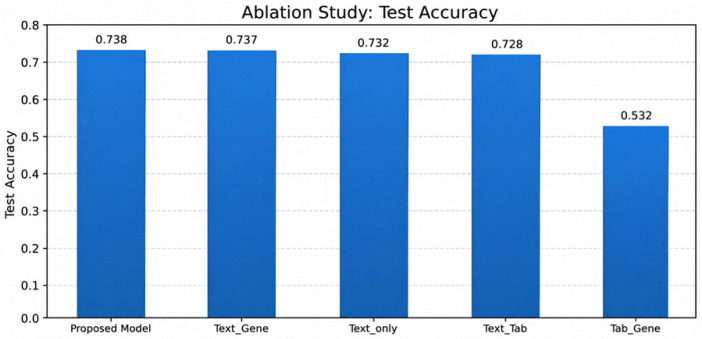
Test accuracy ablation study.

The test accuracy of the ablation study of the proposed full model and combinations of modalities is shown in Figure 8. The best test accuracy overall was obtained by the proposed model with 0.738, a result that shows that the use of ontology-enriched text, tabular metadata, and gene information gives the best overall classification performance. The Text_Gene variant had an accuracy of 0.737, which is very close to the other variants, indicating that gene associations provide complementary information that is useful when combined with disease-level text. The Text_only model was also quite competitive, achieving a test accuracy of 0.732, thereby indicating that textual information is the most powerful single modality for coarse inheritance prediction. The overall accuracy was lower for the Text_Tab variant (0.728), suggesting that tabular metadata is less informative than gene information. The Tab_Gene model, with only non-text modalities, performed far worse (accuracy of 0.532), highlighting that non-text modalities are not enough for accurate inheritance classification. Overall, Figure 8 shows the best test accuracy for the complete multimodal model, with the text being the primary predictive source and the gene information yielding the most valuable complementary contribution.

In Table 11, we present an ablation study of the overfitting phenomenon of the proposed RareFusionBalanced model and its variants by analyzing the training and validation metrics at the optimal obtained checkpoint. The RareFusionBalanced model does not suffer from such severe overfitting, and for the full model, macro-F1 gaps of 0.0282 and 0.0233 exist for seeds 42 and 62, respectively, with limited accuracy gaps and loss gaps. The macro-F1 gap and macro accuracy gap for seed 52 are slightly larger at 0.0560 and 0.0410, respectively, and are considered mild-to-moderate overfitting. However, the validation loss is not that far off from the training loss, indicating that the model has not diverged in an uncontrolled manner from the training loss to the validation loss. The ablation variants also exhibit consistent generalization performance. The Text_only model has a negative macro-F1 gap with a slightly bigger macro-F1 gap on the validation set compared to the training set, and the Text_Tab and Text_Gene models have very small macro-F1 gaps of 0.0034 and 0.0054, respectively. This means that these variants are likely to be suitable for the validation set. The Tab_Gene variant has poor absolute performance, but its small macro-F1 gap and loss gap suggest that its poor performance is primarily due to a lack of predictive power, not overfitting. Table 11 summarizes the overall results, where adopting the suggested regularization method, selective fine-tuning and early stopping were able to prevent severe overfitting in the proposed model and its variants for all but one of the seeds.

Table 12 presents the results of coarse inheritance classification between the proposed RareFusionBalanced and TextOnlyTransformerBalanced. RareFusionBalanced outperforms the text-only transformer baseline on the overall performance, with an accuracy of 0.7382 and macro-F1 of 0.6284, compared with 0.7243 and 0.5795, respectively. The improvement is particularly noteworthy in macro-F1, due to the imbalanced nature of the inheritance classes, so this metric will better serve to appreciate the performance of both the majority and minority classes. The proposed model also demonstrated high mAP and macro-AUC, from 0.6587 to 0.6686 and from 0.9010 to 0.9183, respectively, which demonstrated better class separability and precision–recall behavior. Furthermore, RareFusionBalanced demonstrated improved calibration by achieving a lower ECE of 0.0395 than the text-only model with 0.1194 and a slightly lower Brier-OVR score of 0.0528 than the text-only model with 0.0569. The results indicate that our results are more advantageous than using just text when ontology-enriched disease text is combined with structured metadata and gene-association evidence. The improvements observed are, however, moderate; thus, the impact of the proposed model should be considered primarily in terms of improved multimodal representation, class-balanced performance, and good probability calibration.

## 5. Discussion

RareFusion-Net is not a reasoning system for diagnostic purposes at the patient level, but it is a multimodal benchmark for coarse inheritance classification using curated Orphadata knowledge. In this context, the results show that the proposed approach is able to learn meaningful structure for inheritance from a variety of rare-disease resources by combining knowledge from heterogeneous disease text, structured epidemiological metadata, and gene-association information, which are all enriched with ontological information. RareFusionBalanced achieved an accuracy of 0.7382, a macro-F1 of 0.6284, a micro-F1 of 0.7382, a macro-AUC of 0.9183, and a mAP of 0.6686. The results show that the model contains useful discriminative and ranking information for the seven coarse inheritance categories. Moreover, the expected calibration error of 0.0395 and Brier-OVR score of 0.0528 indicate that the model makes relatively reliable probability estimates that are essential for knowledge-based review and expert-assisted inheritance annotation.

The benefit of multimodal integration is supported by the neural baseline comparison, but the improvement needs to be taken with a grain of salt. On average, the proposed RareFusionBalanced outperformed the TextOnlyTransformerBalanced in terms of accuracy (0.7243), macro-F1 (0.5795), macro-AUC (0.9010), mAP (0.6587), ECE (0.1194), and Brier-OVR (0.0569). These findings indicate that having tabular metadata and gene-association information is helpful and that this extra value is measurable over and beyond the ontology-enriched text information alone. The improvement over strong text-based methods, however, is still low, and the statistical comparison with TFIDF_LinearSVM did not result in a significant difference. The proposed framework should thus not be seen as a definitive, overall best solution to all strong text baselines, but rather as a multimodalized strong baseline with modest performance improvements, calibration benefits, and structured analysis of the effect of various knowledge modalities from rare diseases.

The statistical analysis also demonstrates the robustness and the weakness of the proposed model. Bootstrap analysis showed an accuracy of 0.7382 with a 95% confidence interval of [0.7147, 0.7714] and a macro-F1 of 0.6284 with a 95% confidence interval of [0.5513, 0.6813]. The broader interval of macro-F1 is because of unbalanced multi-class problems—especially where some of the inheritance classes are underrepresented, there is a great amount of instability. From the results of the McNemar test, it was confirmed that the proposed model is effective in learning meaningful structure beyond the trivial prediction of class frequencies, which was the majority baseline. The comparison with the best baseline (TFIDF_LinearSVM) was, however, not statistically significant with McNemar *p*-value = 0.5667. Likewise, the paired bootstrap macro-F1 gain over TFIDF_LinearSVM was 0.0196 with a confidence interval that contained 0. The results show that the model is reliable and well calibrated, with its numerical advantage over the strongest text baseline considered as moderate, rather than statistically significant.

The class-wise classification report indicates that model performance is highest for inheritance categories that are well-supported. The highest class-wise performance was acquired by the algorithm of the Autosomal Recessive class with 0.8042, 0.8313, and 0.8175 scores for precision, recall, and F1 measure, respectively. The other two classes (Unknown/Other with F1-score 0.8128 and X-linked with F1-score 0.7939) performed well, and the acceptable result was obtained for the class Autosomal Dominant with F1-score 0.6878. The model, however, did less well on minority, mixed classes (Autosomal Mixed: 0.3218; Multifactorial: 0.4478). The F1-score of the Mitochondrial class was 0.5000, although this value must be taken with a pinch of salt, as there is very little test support for this class. The results presented here suggest that RareFusion-Net can extract the main patterns of inheritance better than the rare categories, the low-support categories, and the biologically overlapping categories.

The confusion matrix and sample prediction analysis also show the same trend. Predictions for minority categories tend to be more frequently wrong, while Autosomal Recessive, Unknown/Other, and Autosomal Dominant categories are more concentrated around correct predictions. Occasionally, errors are made between AD and AR, suggesting that descriptions at the disease level are not sufficiently discriminative to distinguish among the mechanisms of inheritance of autosomes. The Autosomal Mixed and Multifactorial cases are also challenging, as the term may encompass a variety of biological processes and overlapping inheritance terms. Sample predictions indicate that even cases where a classification is present and unambiguous tend to be correctly predicted, while incorrect predictions are more likely to occur when there is not a clear classification or there is little metadata. This will validate the model for supporting the review of inheritance annotations but cannot be used as a definitive clinical or genetic decision system.

The ROC and the precision–recall analyses offer complementary perspectives on the behavior of the model. The macro-AUC score of 0.9183 is good for one-vs-rest ranking capability for all inherited classes. This indicates that although the hard label prediction is far from perfect, the model can still put the correct class in a high ranking. The mAP of 0.6686 is harder to achieve under class imbalance and indicates that the precision–recall performance is even more constrained for rare and diverse classes. This comes into importance as the distinction is that the ROC-AUC can be high even if the precision and recall for minority classes are less stable. Hence, the results from both ROC and precision–recall indicate that the model possesses useful discriminative capability, and it should be further improved for the recognition of minority classes.

Based on the results of the reliability analysis, the proposed model is reasonably well calibrated. The ECE value was 0.0395, and the reliability diagram indicated that this model curve was within many of the confidence intervals and was close to the ideal diagonal calibration line. This implies that the model’s confidence intervals tend to be consistent with the observed accuracy. The kind of calibration that is relevant for disease-level knowledge-based applications, where the model can be applied in order to prioritize the records for expert review or to highlight uncertain inheritance annotations, will be the ones that are discussed in this section. However, calibration will not resolve the root of the problem in terms of minority class discrimination. It should thus be understood as a complementary strength to enhance the usefulness of the model’s confidence estimates, rather than as evidence that the model is equally useful with all inheritance classes.

The ablation study is a valuable examination of the contribution of each modality. The ablation variants showed the test accuracy of 0.7382 and the test macro-F1 of 0.6284 for the proposed full model, suggesting that the full multimodal configuration provides the best overall performance. The Text_Gene variant also performed very well (test accuracy: 0.7372, macro-F1: 0.6224), indicating that the associations of genes give the best complementary information when used along with ontology-enriched disease text. The test accuracy of the Text_only model was also high (0.7318), and the macro-F1 score was 0.6127, which proved that the Text-only model was highly competitive and disease text was the predominant predictive modality. In comparison, Text_Tab performed slightly lower with test accuracy of 0.5321 and macro-F1 of 0.4041, and Tab_Gene performed much worse with test accuracy of 0.5321 and macro-F1 of 0.3159. These results show that these non-text modalities are not enough to ensure solid inheritance classification and that multimodal benefit relies on a certain degree of selective integration with robust textual representations.

The seed-wise analysis corroborates the overall stability of the proposed model. From seed 42 to 62, test accuracy varied from 0.7350 to 0.7447, suggesting that the results are not significantly affected by the random initialization. Test macro-F1 ranged from 0.6149 to 0.6224, indicating that the model was more sensitive in making the class-balanced prediction than the overall accuracy. The range of the gate values was also relatively consistent across the different seeds: the text gate was set to 1.0000, and the tabular and gene gates were within close tolerances. This indicates that the model is mostly using ontology-enriched text as its primary modality, whereas tabular metadata and gene associations are contributing smaller but persistent complementary signals.

The overfitting analysis demonstrates a successful control over severe overfitting for the revised training strategy. For the entire RareFusionBalanced model, seeds 42 and 62 had slight macro-F1 differences of 0.0282 and 0.0233, respectively, and seed 52 had a larger but still moderate difference of 0.0560. The training behavior was also found to be more stable with the ablation variants, as the gaps between their training and validation were small. Small gaps between training and validation were also observed for ablation variants, reflecting the role of selective transformer fine-tuning, dropout, weight decay, label smoothing, early stopping, and evaluation in improving training behavior. A single run was deemed to have mild-to-moderate overfitting, but there was no severe overfitting in terms of validation-loss divergence. Thus, the new training method is more useful to assess the disease-level benchmark than an irregular training run.

The multitasking and gating components were to be taken with a grain of salt. The objective of the auxiliary tasks is to motivate broader representation learning at the disease level but does not necessarily imply better inheritance classification. Like this, learned gate values indicate what the model internally considers as important for the modalities; however, they are not direct clinical evidence of modality importance. The ablation results give the most reliable interpretation: text enhanced with ontology is the primary source of prediction, associations of genes the most significant secondary source, and tabular metadata the weakest source. So, RareFusion-Net should not be regarded as a biological reasoning system, but rather as a multimodal benchmark and analysis system.

There are some drawbacks to be noted. First, the study is completely disease-level and has no support for patient-level data, family pedigrees, longitudinal phenotypes, or clinical diagnostic workflow. Second, the benchmark is very skewed and estimates of individual classes for low-support classes like Mitochondrial should be used with caution. Third, while the model was tested in several seeds, ablation variants, calibration metrics, and statistical tests, there is a need for independent external testing on other rare-disease resources to confirm wide generalizability. Fourth, the proposed model falls short of the best text baseline, and the improvement could not be replicated in the current test split. Lastly, at the level of single genes and ontology terms, biological interpretability is still limited, and integration with better attribution and pathway-based validation should be considered in the future.

Rare fusion-Net should be regarded as an overall rare-disease-level rare-disease benchmark and multimodal analysis. The key value added by its use is that ontology-enriched biomedical text is the most powerful source for coarse inheritance prediction; carefully curated gene associations and tabular metadata can offer limited, but interpretable, additional contributions. The results also bring into focus the remaining issues of rare-disease-inheritance predictions, such as class imbalance, low-support categories, lack of interpretability, limited performance gain over the top-performing text baseline, and external validation. From these results, we hope the work in the future on selective multimodal fusion, inheritance-specific auxiliary supervision, calibration-aware prediction, and robust independent evaluation on rare-disease knowledge bases can be built.

## 6. Conclusions

In this paper, we introduced RareFusion-Net, an ontology-enriched disease text, structured metadata, and a gene-association-based multimodal framework for the prediction of inheritance of rare disease. RareFusionBalanced achieved a test accuracy of 0.7382, macro-F1 of 0.6284, micro-F1 of 0.7382, macro-AUC of 0.9183, and mAP of 0.6686. The results demonstrate that the framework can acquire meaningful structure related to inheritance from curated sources of rare-disease knowledge. The model also gave a good calibration, with ECE of 0.0395 and Brier-OVR of 0.0528, suggesting that the confidence levels provided by the model are reliable for the purpose of a disease-level knowledge review.

The proposed multimodal model was found to outperform the TextOnlyTransformerBalanced for accuracy, macro-F1, macro-AUC, mAP and calibration. The gain over strong text-based baselines was, however, still modest, and the statistical comparison of the performance of the two models was not significant. The proposed approach thus should be viewed as offering moderate and practically meaningful multimodal advantages, not as a definite superiority over all text-based baselines. It is crucial to underscore this balanced interpretation since ontology-enriched disease text continues to be the top predictive modality in the current benchmark.

Ablation study results demonstrated that the best test accuracy and macro-F1 were obtained by the full model compared to the other models tested, and the Text_Gene configuration yielded very similar results. The results show that gene associations are a source of more valuable complementary information, while tabular metadata is less consistent. The poor results for the Tab_Gene variant indicate that non-text modalities are not sufficient for an accurate inheritance classification. Generally, the model is multimodal, but so long as it is associated with strong representations of text.

The class-wise results show that the model is found to be performing well in major inheritance classes—such as Autosomal Recessive, Unknown/Other, X-linked, and Autosomal Dominant.—but performance is poorer for the Autosomal Mixed, Multifactorial, and Mitochondrial categories due to class imbalance, small sample sizes, and biological heterogeneity. The confusion matrix, precision–recall analysis, and sample predictions also verify that the minority and ambiguous classes are more prone to being mixed with larger inheritance classes. So, the primary unsolved problem is more the improvement of the correct recognition of all categories of inheritance rather than its overall accuracy.

The training and overfitting analysis demonstrated that the regularization and overfitting prevention strategies adopted in this study were able to prevent severe overfitting. Seed-wise evaluation showed the relatively stable accuracy across three random seeds, although macro-F1 was more sensitive to the initialization due to the minority-class behavior. The train–validation gaps were minimal for most neural runs and variants of ablation, and mild-to-moderate overfitting was found for only one seed of RareFusionBalanced. The results confirm the reliability of the revised training procedure in the disease-level benchmark setting.

This work should be viewed as a standard and multimodal analysis model and not as a diagnostic system for a patient. RareFusion-Net aims to help knowledge organization, inheritance annotation review, and hypothesis generation in curated rare disease resources. It is not intended as a replacement for clinical or genetic expertise. It is useful for comparison of possible inheritance patterns, for uncovering potential inconsistencies in curated annotations, and for making calibrated predictions that can aid the expert review process.

Future work should aim at enhancing the generalization of minority classes, the selective fusion mechanism, and the design of auxiliary tasks that are more closely related to the inheritance prediction problem. Other directions are given by external validation on independent resources of rare diseases, class-sensitive calibration, uncertainty-aware rejection mechanisms, gene-level and ontology-level interpretability, repeated evaluation of partition, and finer-grained inheritance modeling. The use of these extensions will be needed to assess the generalizability and practicality of multimodal rare-disease-inheritance prediction.

## Figures and Tables

**Table 1:** State-of-the-arts summary.

Ref	Author	Year	Techniques	Performance Results	Limitations
[17]	Frederiksen et al.	2022	In silico analysis using Orphadata epidemiology, phenotype, gene, natural-history, and linearization files	Showed strong heterogeneity of rare disorders across evidence types	Descriptive rather than predictive
[18]	Sanjak et al.	2024	Ontology-enriched knowledge graph + node embeddings + clustering	Identified semantically coherent rare-disease clusters	Unsupervised; not direct classification
[19]	Moon et al.	2025	Alignment of disease clusters with Orphanet classifications	Connected learned clusters to Orphanet linearized classes	Organizational, not predictive
[20]	Dong et al.	2023	ORDO-based weak supervision for rare-disease identification from clinical notes	Strong Text-to-UMLS stage; lower Text-to-ORDO performance due to mapping errors	Sensitive to ontology matching quality
[21]	Wu et al.	2024	ORDO + UMLS + NLP + LLMs for rare-disease phenotyping	Best F1 around 0.75 with ontology-constrained LLM pipeline	Clinical-note phenotyping only
[22]	Cao et al.	2024	Ontology-enhanced LLMs for automatic rare-disease mining and KG construction	Improved entity/relation extraction over baseline LLMs	Extraction task, not disease-level prediction
[23]	Jia et al.	2018	Phenotype-based rare-disease auxiliary diagnosis	High top-k retrieval accuracy	Early system; limited multimodal fusion
[24]	Alsentzer et al.	2025	Few-shot deep learning over rare-disease knowledge graph	Strong performance on simulated rare-disease patients	Focus on patient-level diagnosis
[25]	Wen et al.	2025	Deep learning for phenotype-driven disease ranking and symptom recommendation	Improved coverage and MRR over baselines	Phenotype-centric, not full Orphadata fusion
[26]	Mohtashamian et al.	2023	Automated ORDO ontology quality assurance	Found many candidates missing relations	QA study, not predictive modeling
[27]	Faviez et al.	2024	Supervised ML + semantic phenotyping for rare-disease EHR detection	Strong AUROC for NPHP1-related ciliopathy identification	Small true-case cohort; extraction limitations

**Table 2:** Raw-to-coarse inheritance mapping rules used in target reformulation.

Raw Inheritance Descriptor Pattern	Coarse Class
autosomal dominant	Autosomal Dominant
autosomal recessive	Autosomal Recessive
autosomal dominant + autosomal recessive in the same descriptor	Autosomal Mixed
x-linked	X-linked
mitochondrial	Mitochondrial
multigenic	Multifactorial
multifactorial	Multifactorial
semi-dominant	Autosomal Dominant
missing	Unknown/Other
No data available	Unknown/Other
unknown	Unknown/Other
not applicable	Unknown/Other
Any remaining unmatched or uncategorized inheritance descriptor	Unknown/Other

**Table 3:** Dataset Distribution.

Class	Train	Validation	Test	Total
Autosomal Recessive	1521	326	326	2173
Unknown/Other	1078	231	231	1540
Autosomal Dominant	1072	230	230	1532
X-linked	328	71	70	469
Autosomal Mixed	204	43	44	291
Multifactorial	136	29	29	194
Mitochondrial	27	6	6	39

**Table 4:** Holdout baseline comparison for coarse inheritance classification.

Model	Accuracy	Macro-F1	Micro-F1	Macro-AUC	mAP	ECE	Brier-OVR
TFIDF_LinearSVM	0.7340	0.6062	0.7340	0.8666	0.6244	0.3505	0.0772
TFIDF_LogReg	0.6709	0.5618	0.6709	0.8720	0.5943	0.1703	0.0704
EarlyFusion_LogReg	0.6122	0.4848	0.6122	0.8700	0.5667	0.1335	0.0780
Tabular_HGB	0.5342	0.3281	0.5342	0.7455	0.3218	0.0905	0.0912
Tabular_RandomForest	0.4370	0.2861	0.4370	0.7095	0.2842	0.1725	0.1045

**Table 5:** Classification report for the proposed model.

Class	Precision	Recall	F1-Score
Autosomal Dominant	0.6680	0.7087	0.6878
Autosomal Mixed	0.3256	0.3182	0.3218
Autosomal Recessive	0.8042	0.8313	0.8175
Mitochondrial	0.5000	0.5000	0.5000
Multifactorial	0.3947	0.5172	0.4478
Unknown/Other	0.8599	0.7706	0.8128
X-linked	0.8525	0.7429	0.7939
Accuracy			0.7382

**Table 6:** Performance of individual random-seed runs for the proposed model.

Seed	Validation Macro-F1	Validation Accuracy	Test Macro-F1	Test Accuracy	Gate Text	Gate Tab	Gate Gene
42	0.6570	0.7489	0.6224	0.7382	1.0000	0.4275	0.3908
52	0.6302	0.7254	0.6149	0.7350	1.0000	0.4108	0.3933
62	0.6839	0.7575	0.6176	0.7447	1.0000	0.4057	0.3960

**Table 7:** Proposed model under different prediction settings.

Prediction Setting	Accuracy	Macro-F1	Micro-F1
Proposed Model	0.7382	0.6284	0.7489
Temperature scaling only	0.7329	0.5948	0.7329
Temperature scaling + bias tuning	0.7329	0.5948	0.7329

**Table 8:** Statistical evaluation of the proposed model.

Metric	Value
Bootstrap Accuracy	0.7382
95% CI for Accuracy	[0.7147, 0.7714]
Bootstrap Macro-F1	0.6284
95% CI for Macro-F1	[0.5513, 0.6813]
Bootstrap Micro-F1	0.7382
95% CI for Micro-F1	[0.7147, 0.7714]
McNemar statistic vs. majority baseline	283.6688
McNemar *p*-value vs. majority baseline	0.0000
McNemar statistic vs. strongest baseline (TFIDF_LinearSVM)	0.3282
McNemar *p*-value vs. strongest baseline (TFIDF_LinearSVM)	0.5667
Paired bootstrap Macro-F1 gain vs. TFIDF_LinearSVM	0.0196
95% CI for Macro-F1 gain vs. TFIDF_LinearSVM	[−0.0246, 0.0655]
Mean one-vs-rest Brier score after temperature scaling	0.0528
Expected Calibration Error (ECE)	0.0395

**Table 9:** Sample predictions of the proposed model for the main inheritance task.

OrphaCode	Disease Name	Age of Onset	Prevalence Type	Geographic Prevalence	True Coarse Inheritance	Predicted Coarse Inheritance	Result
2324	Osteopenia-intellectual disability-sparse hair syndrome	Infancy, Neonatal	Cases/families	Worldwide	Autosomal Recessive	Autosomal Recessive	Correct
83617	Agammaglobulinemia-microcephaly-craniosynostosis-severe dermatitis syndrome	Antenatal, Infancy, Neonatal	Cases/families	Worldwide	Autosomal Recessive	Autosomal Recessive	Correct
1231	Barber–Say syndrome	Neonatal	Cases/families	Worldwide	Autosomal Mixed	Autosomal Dominant	Incorrect
320396	Autosomal recessive spastic paraplegia type 45	Infancy	Cases/families	Worldwide	Autosomal Recessive	Autosomal Recessive	Correct
48918	Focal myositis	Adult	Cases/families	Worldwide	Unknown/Other	Unknown/Other	Correct
314575	Intellectual disability-hypotonia-brachycephaly-pyloric stenosis-cryptorchidism syndrome	Infancy, Neonatal	Cases/families	Worldwide	Autosomal Recessive	Autosomal Recessive	Correct
85327	X-linked intellectual disability-acromegaly-hyperactivity syndrome	Childhood	Cases/families	Worldwide	X-linked	X-linked	Correct
97364	Bilateral multicystic dysplastic kidney	Antenatal, Neonatal	Point prevalence	Worldwide	Autosomal Dominant	Autosomal Dominant	Correct
163956	X-linked intellectual disability, Nascimento type	Infancy	Cases/families	Worldwide	X-linked	X-linked	Correct
1202	Larynx atresia	All ages	Point prevalence	Worldwide	Autosomal Dominant	Unknown/Other	Incorrect
694356	ADAR-related hereditary spastic paraplegia	Nan	NaN	NaN	Autosomal Dominant	Autosomal Recessive	Incorrect
401780	Autosomal recessive spastic paraplegia type 61	Infancy	Cases/families	Worldwide	Autosomal Recessive	Autosomal Recessive	Correct
3163	SHORT syndrome	Neonatal	Cases/families	Worldwide	Autosomal Dominant	Autosomal Recessive	Incorrect
1037	Arthrogryposis multiplex congenita	Neonatal	Prevalence at birth	Europe	X-linked	X-linked	Correct
139518	Distal hereditary motor neuropathy type 1	Adolescent, Adult, Childhood	NaN	NaN	Autosomal Dominant	Autosomal Dominant	Correct

**Table 10:** Ablation study summary.

Model	Val Accuracy	Val Macro-F1	Test Accuracy	Test Macro-F1	Test Micro-F1	Gate Text	Gate Tab	Gate Gene
**Proposed Model**	0.7489	0.6570	0.7382	0.6284	0.7382	1.0000	0.4275	0.3908
**Text_Gene**	0.7532	0.6716	0.7372	0.6224	0.7372	1.0000	0.0000	0.3896
**Text_only**	0.7436	0.6603	0.7318	0.6127	0.7318	1.0000	0.0000	0.0000
**Text_Tab**	0.7489	0.6520	0.7276	0.6087	0.7276	1.0000	0.4162	0.0000
**Tab_Gene**	0.5556	0.3618	0.5321	0.3159	0.5321	1.0000	0.4410	0.4097

**Table 11:** Overfitting summary for the neural models and ablation variants.

Experiment	Seed	Best Epoch	Train Macro-F1	Val Macro-F1	Macro-F1 Gap	Train Accuracy	Val Accuracy	Accuracy Gap	Train Loss	Val Loss	Loss Gap	Overfitting Status
RareFusionBalanced	42	26	0.6852	0.6570	0.0282	0.7721	0.7489	0.0242	0.3000	0.3677	0.0677	No severe overfitting
	52	26	0.6862	0.6302	0.0560	0.7643	0.7233	0.0410	0.2985	0.3704	0.0719	Mild-to-moderate overfitting
	62	26	0.7072	0.6839	0.0233	0.7753	0.7575	0.0178	0.2963	0.3565	0.0602	No severe overfitting
Text_only	42	20	0.6483	0.6603	−0.0120	0.7359	0.7436	−0.0077	0.3336	0.3776	0.0440	No severe overfitting
Text_Tab	42	22	0.6554	0.6520	0.0034	0.7501	0.7489	0.0012	0.3182	0.3621	0.0439	No severe overfitting
Text_Gene	42	25	0.6771	0.6716	0.0054	0.7652	0.7532	0.0120	0.3103	0.3750	0.0648	No severe overfitting
Tab_Gene	42	23	0.3758	0.3618	0.0140	0.5460	0.5556	−0.0095	0.5155	0.5353	0.0198	No severe overfitting

**Table 12:** Neural model performance comparison for the coarse inheritance classification task.

Model	Temperature	Accuracy	Macro-F1	Micro-F1	Macro-AUC	mAP	ECE	Brier-OVR
RareFusionBalanced	1.0000	0.7382	0.6284	0.7489	0.9183	0.6686	0.0395	0.0528
TextOnlyTransformerBalanced	1.0000	0.7243	0.5795	0.7243	0.9010	0.6587	0.1194	0.0569

## Data Availability

https://www.kaggle.com/datasets/ahsanneural/rare-diseases-orphadata-2026, accessed on 1 March 2026, and Source Code available on https://github.com/KOkab2020/RareDisease/commits/RareDisease, accessed on 1 March 2026.
